# Correction to “Beyond Infections: A Case Report on the Identification of Neuroleptic Malignant Syndrome (NMS) in a Complex Clinical Context”

**DOI:** 10.1002/ccr3.71717

**Published:** 2025-12-22

J. Wang and Y. Yang, “Beyond Infections: A Case Report on the Identification of Neuroleptic Malignant Syndrome (NMS) in a Complex Clinical Context,” *Clinical Case Reports* 13, no. 10 (2025): e71333, https://doi.org/10.1002/ccr3.71333.

“Madopar” was incorrectly referred to as “Memantine” in the following sections of the article: “key words,” “abstract,” and “Case presentation.” Where “Memantine” was mistakenly written, it should be replaced with “Madopar.” Please see further details below.

In the section “key words,” the word “Memantine” was incorrect. It should be replaced with “Madopar.”

In the “abstract,” the text “the discontinuation of memantine and amantadine” was incorrect. This should have read “the discontinuation of madopar and amantadine.”

In the “Case presentation,” the text “The patient had used memantine and amantadine for anti‐Parkinson treatment for a long time” and “In such an instance, physicians decided to stop amantadine and memantine to see if the symptoms subsides” was incorrect. This should have read “The patient had used madopar and amantadine for anti‐Parkinson treatment for a long time” and “In such an instance, physicians decided to stop amantadine and madopar to see if the symptoms subsides.”

We apologize for this error.

